# Dual Role of HIV-1 Envelope Signal Peptide in Immune Evasion

**DOI:** 10.3390/v14040808

**Published:** 2022-04-13

**Authors:** Chitra Upadhyay, Priyanka Gadam Rao, Roya Feyznezhad

**Affiliations:** Division of Infectious Disease, Department of Medicine, Icahn School of Medicine at Mount Sinai, 1 Gustave L. Levy Place, New York, NY 10029, USA; priyanka.rao@mssm.edu (P.G.R.); feyz001@gmail.com (R.F.)

**Keywords:** HIV-1, envelope, glycosylation, Vpu, tetherin antagonism, antibodies, DC-SIGN, transmission

## Abstract

HIV-1 Env signal peptide (SP) is an important contributor to Env functions. Env is generated from Vpu/Env encoded bicistronic mRNA such that the 5′ end of Env-N-terminus, that encodes for Env-SP overlaps with 3′ end of Vpu. Env SP displays high sequence diversity, which translates into high variability in Vpu sequence. This study aimed to understand the effect of sequence polymorphism in the Vpu-Env overlapping region (VEOR) on the functions of two vital viral proteins: Vpu and Env. We used infectious molecular clone pNL4.3-CMU06 and swapped its SP (or VEOR) with that from other HIV-1 isolates. Swapping VEOR did not affect virus production in the absence of tetherin however, presence of tetherin significantly altered the release of virus progeny. VEOR also altered Vpu’s ability to downregulate CD4 and tetherin. We next tested the effect of these swaps on Env functions. Analyzing the binding of monoclonal antibodies to membrane embedded Env revealed changes in the antigenic landscape of swapped Envs. These swaps affected the oligosaccharide composition of Env-N-glycans as shown by changes in DC-SIGN-mediated virus transmission. Our study suggests that genetic diversity in VEOR plays an important role in the differential pathogenesis and also assist in immune evasion by altering Env epitope exposure.

## 1. Introduction

**Figure 1:**
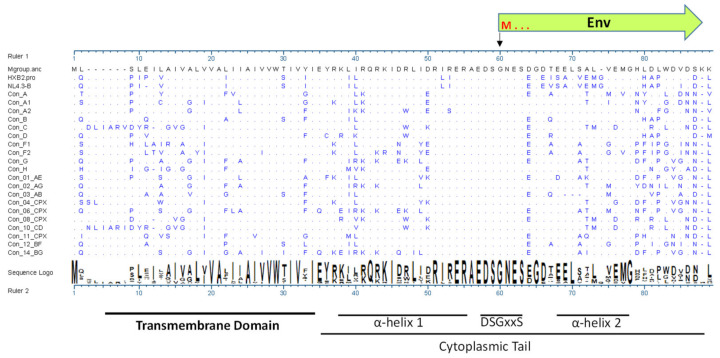
Alignment of consensus sequence of Vpu from HIV-1 group M subtypes showing amino acid variability especially in the C-terminus. Source: Los Alamos HIV Database at https://www.hiv.lanl.gov/content/sequence/HIV/mainpage.html (accessed on 1 January 2022). The first amino acid in Env open reading frame methionine is denoted by M.

Efficiency of HIV-1 infection and subsequent disease progression are determined by various host-pathogen interactions. As host responses can block virus replication, HIV-1 has developed various strategies to counteract or evade these antiviral defense mechanisms mainly via the viral accessory proteins including the Vif, Vpr, Vpu, and Nef proteins. These accessory proteins can be dispensable for viral replication in vitro but are essential for modulating the host cellular environment to promote efficient viral replication, transmission, and evasion from innate and acquired immunity. The Vpu gene is present in HIV-1 but not in HIV-2 and other related SIVs, such as SIV from sooty mangabey and SIV from rhesus macaques [1]. Acquiring the anti-tetherin function by Vpu is suggested to be important for making the transition into human hosts [2]. Vpu is translated from a bicistronic mRNA that also encodes the HIV-1 envelope (Env) gene. The 3′ end of Vpu, herein Vpu C-terminus (C-term), overlaps with 5′ N-terminus of Env that encodes, from a different reading frame, ~30 amino acids (aa) of the Env signal peptide (SP). Thus, during virus replication, Vpu and Env are produced late and in a coordinated manner [3]. One remarkable feature of this gene segment is its sequence variability, which translates into a high degree of aa diversity for both Vpu and Env (Figure 1 and [4]).

The HIV-1 accessory protein Vpu is assigned multiple critical functions including (1) down-modulate CD4 receptor to prevent its interaction with HIV-1 Env and facilitate its incorporation into the virions; (2) antagonize host restriction factor tetherin (BST2) to allow the release of progeny virions from infected cells; and (3) suppress the induction of interferon-I (IFN-I) response via multiple mechanisms dependent on tetherin or other host factors [2,5,6,7]. The Vpu proteins from HIV-1 isolates share about 35% sequence identity, with high degree of similarity in the transmembrane and α-helix 1, while relatively high variability in the α-helical region 2 and the C terminus. The C-terminal end of HIV-1 Vpu harbors a clade specific determinant that antagonizes tetherin and facilitates virion release [8,9]. This region also harbors a motif necessary for down-regulating CD1d from the surface of infected dendritic cells (DCs), thus inhibiting their crosstalk with the innate invariant natural killer T (iNKT) cells [10,11]. Furthermore, signature residues in the C-terminus of Vpu are associated with NK cell escape in KIR2DL2 positive individuals [12]. Thus, Vpu C-terminus is involved in important functions that assist in viral evasion from innate immunity.

HIV-1 Env and its SP are under immune pressure incorporating changes that may stabilize the Env spike, enhance Env’s interaction with host cells and also assist in dodging the interaction with Abs. Comparing the SP sequence among the transmitted-founder (T/F), acute and chronic isolates show insertion and deletions of residues [13], the significance of which is unclear. The HIV-1 Env biogenesis initiates in the endoplasmic reticulum (ER) where the nascent proteins remain tethered via its SP until the Env attains conformational folding, glycosylation, and di-sulfide bonds formation [14,15]. Thereafter, the SP is cleaved allowing the native trimer to egress from the ER to Golgi apparatus where further glycan maturation takes place. Thus, SP is not a part of the mature Env that is displayed on the virions and on the infected cells.

The HIV-1 Env, made of surface subunit gp120 and transmembrane subunit gp41, is indispensable for initiating virus infection. Env is the sole viral protein exposed to host antibody response and is the main focus for vaccine development. One extraordinary property of the HIV-1 Env is its extreme level of glycosylation. HIV-1 Env has 18–33 potential N-linked glycosylation sites (PNGS) [16,17,18]; the majority of glycans are found on gp120. All three types of N-glycans, high mannose, hybrid, and complex, are displayed [19]. The glycans play a critical role in determining the conformation of the Env trimers and the ability of the virus to counteract host antibody responses. These glycans facilitate immune evasion by masking the neutralizing antibody (NAb) epitopes [20] and also form epitopes for broadly neutralizing PG9/16 and PGT (bNAbs) [21,22,23,24,25,26,27,28]. They also promote HIV-1 infection and transmission via DC-SIGN and other mannose-binding proteins [29]. While the mechanisms that control the heterogeneity of N-glycan patterns and composition are not fully understood our studies have shown that amino acids in the Env SP can control the Env glycan composition which in turn alters the Env-Ab interactions [4,30]. Thus, Env-SP plays an important role in immune evasion by altering recognition by antibodies.

This study was aimed to investigate the biologic importance of this element (Vpu-Env overlapping domain (VEOR)) that impacts the function of two vital proteins (i.e., Vpu and Env). We recently published the impact of VEOR, as Env SP, on Env glycosylation, functions, and virus phenotype. We swapped the VEOR of infectious molecular clone (IMC) pNL-CMU06 (WT) with VEOR from other HIV-1 isolates (MW965.26, 398F1, CH119 and 271.1). In HEK293T cells, which lack endogenous tetherin, the swap had no effect on level of produced virions, or infectivity of the released virions, although expression of Env and Vpu protein was affected. In contrast, virion release and infectivity of viruses produced from 293T cells in presence of tetherin and in TZM.bl cells that express endogenous tetherin was affected. When VEOR of MW965.26 was planted onto Env from other isolates similar reduction in virus release (in presence of tetherin) was observed. When the impact of this overlapping region was evaluated in the context of Env-SP, altered Env glycosylation and Env interaction with monoclonal antibodies (mAbs) targeting V2, V3, and gp41 was observed [30]. In addition, swaps also incurred changes in virus transmission via C-type lectin DC-SIGN. These results show significant changes in Vpu and Env functions as a result of amino acid changes in the VEOR. Further studies to understand the mechanistic connection of VEOR to HIV-1 pathogenesis are warranted.

## 2. Materials and Methods

### 2.1. Plasmids, Cell Culture and Viruses

Generation of pNL4.3-CMU06, pNL-SF162, pREJO, and its SP variant IMCs has been previously described [30]. The SPs and Env were selected to represent isolates that display different neutralization sensitivity and represent different clades and clinical stages. The MW SP was selected based on its high neutralization sensitivity; it is one of the most neutralization sensitive HIV-1 isolate known. The SP from CH119, 271 and 398F1 were selected as these isolates comprise the global panel of HIV-1 (PMID: 24352443) while 271 was picked randomly (PMID: 19939925).

**Figure 2:**
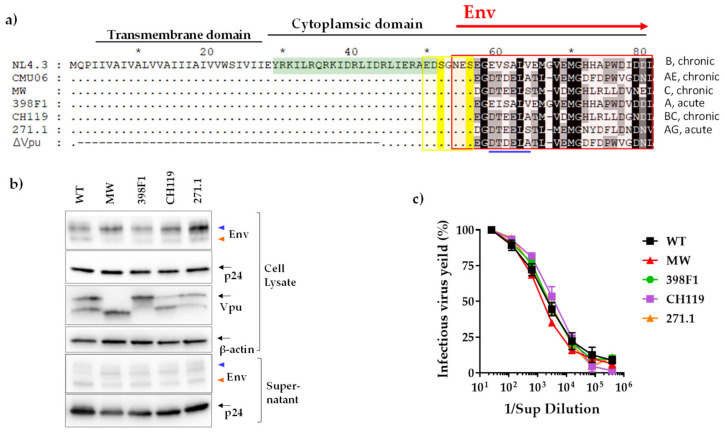
Effect of Vpu C-term swaps. (**a**) Amino acid alignment with reference to NL4.3. Similarity is shown by dot, insertion/deletion by trace, *, denotes blocks of 10. Important functional domains are indicated above the sequences. The canonical sorting motif (ExxxLV) is shown by blue line and the di-serine motif is highlighted in yellow. The region designated as C-terminus (VEOR) in this study is enclosed in red box. ΔVpu is CMU06 with deleted Vpu region shown in traces, similarity is shown in dots. Vpu region highlighted in green was used to immunize rabbit for producing polyclonal Ab. Infectious viruses were produced by transfecting 293T cells, that lack tetherin, with infectious molecular clones (IMC) of CMU06 proviral construct harboring chimeric Vpus and were assessed for (**b**) protein expression and (**c**) virus titer. The anti-gp120 MAb cocktail detected two Env bands corresponding to gp120 and gp160 shown by orange and blue triangle, respectively. β-actin was used as loading controls for cell lysates.

CMU06ΔVpu IMC was generated by deleting the transmembrane and part of cytoplasmic domain of the Vpu (Figure 2a). Plasmids expressing human tetherin and CD4 were obtained from Frank Kirchhoff and are described elsewhere [2].

HEK293T cells were obtained from the American Type Culture Collection (ATCC, Manassas, VA, USA). The following reagents were obtained through the NIH AIDS Reagent Program, Division of AIDS, NIAID, NIH: TZM-bl from Dr. John C. Kappes, Dr. Xiaoyun Wu and Tranzyme Inc (San Diego, CA, USA) [31]; Raji and Raji/DC-SIGN cells from Dr. Li Wu and Dr. Vineet N. KewalRamani [32]. All cell lines were maintained in Dulbecco’s modified Eagle medium (DMEM) supplemented with 10% heat-inactivated fetal bovine serum (FBS), HEPES pH 7.4 (10 mM), L-glutamine (2 mM), penicillin (100 U/mL), and streptomycin (100 μg/mL) at 37 °C in a humidified atmosphere with 5% CO_2_. Transfection of cells was performed using JetPEi (Polyplus, New York, NY, USA) reagent as per the manufacturer’s instructions.

To generate viruses, HEK293T cells were transfected with wild type (WT) or swapped pNL-CMU06 plasmids using jetPEI transfection reagent (Polyplus, New York, NY, USA). Supernatants were harvested after 48 h. and clarified by centrifugation and 0.22 μm filtration. Single-use aliquots were stored at −80 °C. Virus infectivity was assessed on TZM.bl cells as described [4,30,33]. An HIV-1 p24 enzyme-linked immunosorbent assay kit (Takara, San Jose, CA, USA) was used to quantify the p24 content in supernatants using the manufacturer’s protocols. To generate PBMC-derived viral stocks, PBMC were isolated by lymphoprep gradient centrifugation from leukopaks of HIV-1 seronegative donors (New York Blood Center, New York, NY, USA). Prior to HIV-1 infection, PBMC were activated by incubation in RPMI-intereukin-2 (IL-2) growth medium containing 10 μg of phytohemagglutinin (PHA) (PHA-P) per ml. The RPMI-IL-2 growth medium was RPMI 1640 medium supplemented with 10% heat-inactivated fetal calf serum, HEPES pH 7.4 (10 mM), L-glutamine (2 mM), penicillin (100 U/mL) and streptomycin (100 μg/mL) and 20 U of recombinant IL-2 per mL. After overnight incubation with PHA, cells were washed and cultured with IL-2 for additional 2 to 3 days. All cultures were maintained in 5% CO_2_ incubators at 37 °C. PBMCs were exposed to virus (50 ng p24 of 293T-derived stock) overnight and then washed to remove the viral inoculum. Virus infectivity was measured in TZM.bl cell line as above and p24 contents were measured by p24 ELISA kit (Takara, San Jose, CA, USA) as per the manufacturer’s instructions.

### 2.2. Western Blot

To monitor expression of Env, p24 and Vpu, HEK293T cells were transfected with CMU06 IMC in 6-well plate. Two days post-transfection, supernatant and cells were harvested. Supernatant were clarified and virus particles were pelleted using sucrose cushion [4]. Cells and sucrose-pelleted viruses were lysed and separated in 4 to 12% Tris-HCl gels (Bio-Rad, Hercules, CA, USA). After gel electrophoresis, proteins were transferred onto nitrocellulose membranes and reacted with gp120 mAb cocktail (Env), gag-p24 (91-5D) [4,30], and Vpu polyclonal antibody produced in rabbit (Genscript, Piscataway, NJ, USA). Subsequently, blots were probed with anti-human (Env and p24) and anti-rabbit (Vpu) HRP labelled secondary Ab. HRP anti-β-actin antibody (BioLegend, San Diego, CA, USA) was used to as loading control in cell lysates. Proteins were detected using Clarity Western ECL Substrate (Bio-Rad, Hercules, CA, USA) and ImageLab Bio-Rad scanner.

### 2.3. Tetherin Antagonism

To determine the capability of Vpu to antagonize tetherin, HEK293T cells were seeded in 48-well plates and transfected with 200 ng of CMU06 IMCs, and different dilutions of tetherin expression plasmid. At 2 days post-transfection, supernatants were harvested, and the yield of infectious HIV-1 was determined by a 96-well infection assay on TZM-bl indicator cells as described previously [4,30]. TZM.bl cells were transfected with IMCs alone as these cells express endogenous tetherin and the yield of infectious virus was determined as above.

### 2.4. Flow Cytometry

To determine the effect of Vpu on cell surface expression of tetherin and CD4, TZM.bl cells, expressing tetherin and CD4, were transfected with IMCs. 48 h post transfection cells were separately stained for surface tetherin (allophycocyanin (APC)-conjugated anti-human tetherin antibody from BioLegend, San Diego, CA, USA) and CD4 (APC-conjugated anti-human CD4 from eBiosciences, San Diego, CA, USA), as per the manufacturer’s instruction. Cells were analyzed by Attune flow cytometer, and >100,000 events were collected. Analysis was carried out using FCS-Express software as follows: TZM.bl cells were selected from a plot of forward-area vs. side scatter-area (FSC-A/SSC-A) from which doublets were excluded in a forward scatter height vs. forward scatter area plot (FSC-H/FSC-A). Live cells were selected by Aqua-negative gating, and geometric mean fluorescent intensity (MFI) of APC+ cells, representing anti-CD4 or anti-tetherin stained cells, were quantified. Background MFI, as determined from cells stained with APC isotype control was subtracted. Vpu-mediated tetherin or CD4 down-modulation is presented as median fluorescence intensity (MFI).

### 2.5. Human Monoclonal Antibodies

The following antibody reagents were obtained through the NIH HIV Reagent Program (https://www.hivreagentprogram.org/Home.aspx accessed on 1 January 2022), Division of AIDS, NIAID, NIH: Anti-HIV-1 gp120 monoclonal PGT121, PGT128 from IAVI [34]; anti-HIV-1 gp120 monoclonal CH59 from Dr. Barton F. Haynes and Dr. Hua-Xin Liao [35]; anti-HIV-1 gp120 monoclonal 3BNC117 from Dr. Michel C. Nussenzweig [36]; anti-HIV-1 gp120 monoclonal 17b from Dr. James E. Robinson [37]; anti-HIV-1 gp120 monoclonal 2G12 from Polymun Scientific, Klosterneuburg, Austria [38]; anti-HIV-1 gp41/gp120 monoclonal 35O22, from Dr. Jinghe Huang and Dr. Mark Connors [38]; anti-HIV-1 gp41/gp120 monoclonal PGT151 from Dennis Burton; anti-HIV-1 gp41 monoclonal 4E10 from Polymun Scientific, Klosterneuburg, Austria [39]. The V2i, V3, gp41 mAbs were produced in our laboratory as described [24,40,41,42,43,44,45,46]. An irrelevant anti-anthrax mAbs 3685 were used as negative controls.

### 2.6. Cell-Associated Env Binding Assay

Assay to detect antibody binding to cell surface expressed Env was performed as described [30]. Briefly, 4 × 10^6^ 293T cells seeded in 100-mm tissue culture dish were transfected with 20 μg gp160 expression plasmid (WT or swapped) using JetPEI (Polyplus, New York, NY USA) (DNA:JetPEI ratio of 1:3) following manufacturer’s instructions. The transfected cells were incubated for 24 h at 37 °C, washed with PBS, detached with trypsin-free cell-dissociation buffer, and resuspended in PBS containing 2% BSA. Cells were stained with Live/dead Aqua stain and distributed into 96-well tissue culture plates (5 × 10^4^/well) for individual staining reactions. Cells were incubated with mAbs at concentrations detailed in figure legends. For detection of mAb binding, biotinylated goat anti-Human IgG Fc (1:1000) followed by streptavidin phycoerythrin (PE) (1:500) was used. The cells were washed 3× with PBS-B (PBS plus 1%BSA) after each step and all incubation steps were performed on ice for 30 min. Cells were analyzed with a BD Fortessa flow cytometer (Becton Dickinson, Carlsbad, CA, USA) and 30,000 events were collected in the PE+ gate. Analysis was carried out using FCS-Express software as follows: HEK293T cells were selected from a plot of forward-area vs. side scatter-area (FSC-A/SSC-A) from which doublets were excluded in a forward scatter height vs. forward scatter area plot (FSC-H/FSC-A). Live cells were selected by Aqua-negative gating, and geometric mean fluorescent intensity (MFI) of PE+ cells, representing anti-Env-stained cells, were quantified. Background MFI, as determined from cells stained with non-HIV-1 mAb (anti-parvovirus mAb 3685) was subtracted from all Env-mAb pairs. Normalized delta MFI (WT-swapped) is shown for CMU06 viruses and normalized MFI with WT set as 100% are shown for REJO and SF162.

### 2.7. DC-SIGN-Mediated Virus Transmission Assays

DC-SIGN mediated transmission assays were performed as described before [4]. Briefly, Raji or DC-SIGN+ Raji cells (1 × 10^5^) were incubated for 2 h with virus (3 ng/mL p24), washed three times to remove unbound virus and co-cultured with TZM-bl cells for 48 h in the presence of DEAE-dextran. HIV-1 transmission to TZM.bl cells was quantified by measuring β-galactosidase activity (Promega, Madison, WI, USA). Percent transmission normalized to infectivity of each virus in TZM.bl cells in the absence of DC-SIGN (set as 100%) are shown.

### 2.8. Statistical Analysis

Statistical calculations were performed using one-way or two-way ANOVA test using GraphPad Prism version 9.1.2 (GraphPad, San Diego, CA USA).

## 3. Results

### 3.1. HIV-1 Vpu with C-Terminal Swaps Have Altered Vpu Expression and Tetherin Antagonism

To examine the effects of (VEOR) on Vpu functions including tetherin antagonism, we generated infectious molecular clone (IMC) of CMU06 with swapped VEOR (Vpu C-term and Env SP) [30]. The VEOR of CMU06 (CRF01_AE, tier 2, acute) was replaced with that of MW965.26 (clade C, tier 1A, chronic), 398F1 (clade A, tier 1A, acute), CH119 (CRF_07, tier 2, chronic), and 271 (clade C, tier 2, acute). The selection was made to represent isolates that display different neutralization sensitivity, different clades and clinical stages (Figure 2a) [47,48,49]. First, we examined the effect of VEOR, on viral protein (Vpu, p24 and Env) expression and virus progeny production in the absence of tetherin, by transfecting HEK293T cells with the IMC’s (Figure 2a–c). When probed with anti-gp120 MAbs, two Env bands (lower:120kDA and upper:160 kDa), were observed in both virus and cell lysates. While the VEOR swaps did not affect virus titer (Figure 2c) changes in expression of Env and p24 were observed compared to the WT (Figure 2b and [30]). The swaps also altered the expression of the upper and lower band compared to the WT and these changes were evident in both cell lysate and supernatant. The differentially processed Env (gp120 vs. gp160) on virions may act as antigenic decoy, helping the virus to better evade the Ab response. Different Env forms on virions are indeed suggested to confer a fitness advantage to the virus by diverting the antibody response towards non-neutralizing epitopes and helping the virus to evade neutralization [50]. When probed with polyclonal Vpu Ab two bands were observed in WT, CH119 and 271.1 while MW only presented the lower band and only the upper band was detected in 398F1. We do not know the reason for the appearance of the double bands, but the region targeted by the Ab used to probe Vpu was similar in all of the VEOR swaps (Figure 2a).

**Figure 3:**
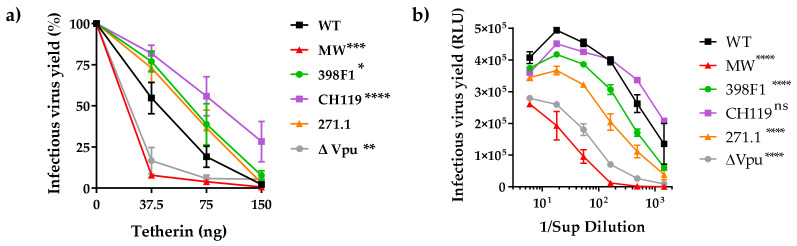
Effect of Vpu swaps on virus release in presence of tetherin. (**a**) Virus release from 293T cells following co-transfection with IMCs in presence of various amounts of plasmid expressing human tetherin. Release of infectious virus was determined by infection of TZM-bl indicator cells and is shown as percentage of the release efficiency in the absence of tetherin, set at 100%. Infections were performed in triplicate and mean and SEM from three experiments are shown *, *p* = 0.03; **, *p* = 0.002; ***, *p* = 0.0002; ****, *p* < 0.0001 by two-way ANOVA vs. WT. (**b**) Virus release from TZM.bl cells, expressing endogenous tetherin, following transfection with proviral IMC bearing the chimeric Vpu and Env. Virus release was assessed by infecting TZM.bl cells with the supernatant from each transfection. Mean +SEM from representative experiment performed in duplicate are shown. ****, *p* < 0.0001 by two-way ANOVA.

We next examined the effects of these chimeric IMCs on the efficiency of virus release from HEK293T cells in presence of tetherin. We co-transfected HEK293T cells with CMU06 IMCs together with plasmid expressing human tetherin at different concentrations (Figure 3a). The results showed that MW VEOR completely disrupted the ability of Vpu to counteract tetherin resulting in abolished virus release (Figure 3a), an effect also seen with the CMU06ΔVpu that was used as control. Notably, expression of MW Vpu was strikingly different, expressing a lower molecular weight Vpu protein compared to the WT (Figure 2b). Presumably, the lower protein band is non-functional or has reduced functionality for antagonizing tetherin and may account for the abrogation for virus release (Figure 3a). The MW VEOR swap also altered the putative trafficking motif (EXXXLV) in the second alpha helix of the Vpu cytoplasmic tail, suggested to be required for efficient tetherin antagonism [51]. However swapping VEOR with 271.1 also incurred changes in the EXXXLV motif identical to MW (Figure 2a) which maintained functional tetherin antagonism, implying involvement of other VEOR residues and/or host factors with reduced virus release. In contrast, 398F1, CH119 and 271.1 VEOR improved the ability of Vpu to enhance virus release. The expression of Vpu protein in these three clones was also different compared to the WT (Figure 2b). The CH119 Vpu expression was lower compared to WT, yet the Vpu was functionally more efficient compared to WT and other VEORs.

To examine the effects of VEOR on the efficiency of virus release in context of physiological concentration of tetherin we transfected TZM.bl cells with equal amounts of chimeric CMU06 IMCs (Figure 3b). At 48 h post-transfection, supernatants containing virus was collected and virus release were quantified by measuring infectivity in TZM.bl cells. As expected, virus release from TZM.bl cells were dependent on Vpu, and we observed 31% reduction in virion release from TZM.bl cells transfected with ΔVpu compared to WT. Chimeric IMC with MW VEOR similarly had decreased virus yield (36%) vs. WT, although virus release was not completely abrogated as seen in Figure 3a suggesting that lower tetherin concentration are present in TZM.bl cells or other virus-host factors work in concert allowing the release of virus albeit at low levels. The other three VEORs (398F1, CH119 and 271.1) also had relatively lower virus yield compared to WT, a pattern different from that observed in HEK293T cells (Figure 3a). Notably, TZM.bl cells are reporter HeLa cells modified to express HIV-1 receptor (human CD4) and co-receptors (CCR5 and CXCR4) [31]. Prior studies have shown that CD4 expression can also reduce the infectivity of released virions by sequestering of the viral envelope by CD4 [52,53].

**Figure 4:**
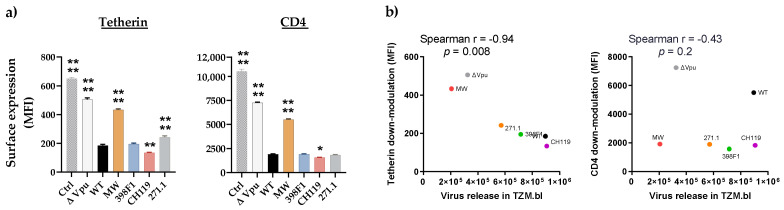
Down modulation of tetherin and CD4. (**a**) TZM.bl cells expressing endogenous tetherin and CD4 were transfected with CMU06 proviral plasmids expressing chimeric Vpu and Env. Cells were analyzed for tetherin and CD4 expression 48h post-transfection by flow cytometry. Median fluorescent intensity (MFI) + SD values of triplicates from representative experiment are shown. *, *p* <0.05; **, *p* < 0.01; ****, *p* < 0.0001 by one-way ANOVA vs. WT. (**b**) Spearman correlation between downmodulation of surface tetherin or CD4 and virus release in TZM.bl cells (AUC from infectivity curves in Figure 3).

In addition to tetherin, Vpu is also known to down-modulate the expression of CD4 to prevent its interaction with HIV-1 envelope (Env) facilitating its incorporation into the virions. To assess the impact of VEOR on Vpu’s ability to down-regulate surface human tetherin and CD4, we transfected TZM.bl cells, expressing endogenous CD4 and tetherin, with IMCs expressing the chimeric Vpu. Compared to WT, CH119 (MFI: 134) had significantly greater ability of downmodulating cell surface tetherin expression (Figure 4a, left panel), while 398F1 (MFI: 195) was comparable to WT (MFI: 185). Contrariwise, efficiency of Vpu with 271.1 VEOR to downregulate tetherin was reduced vs. the WT. As expected, the ΔVpu (MFI: 505) and MW (MFI: 433) were less efficient in reducing tetherin cell surface expression compared to WT. Of note, all IMCs differ only in their VEOR and otherwise have identical sequences, including Nef another accessory protein also involved in tetherin and CD4 antagonism [52,53,54,55,56,57]. Overall, a strong inverse correlation was observed between surface tetherin downmodulation and virion release (Figure 4b). When CD4 down-modulation was assessed, MW and ΔVpu were less efficient while CH119 had better ability to reduce cell surface expression of CD4. (Figure 4, right panel). No correlation existed between CD4 downmodulation and release of virus progeny, suggesting the involvement of factor other than Vpu for the observed lower virion release in TZM.bl vs. HEK293T.

While CD4 downmodulation is a function of Vpu, Env sequestration by CD4 relies on the interaction between the Env and CD4. While all CMU06 viruses in this study had identical Env encoding regions each Env varied phenotypically which may account for the differences observed in relatively different levels of CD4 down-regulation. Notably, the Trp22 in the transmembrane domain of Vpu is critically required for Vpu-mediated downregulation of CD4 [58], which is present in all variants tested and is out of the swapping domain (Figure 2a). These findings highlight the exploitation of other mechanisms by HIV-1 to downregulate CD4 and thus promote viral pathogenesis and warrants further investigations.

**Figure 5:**
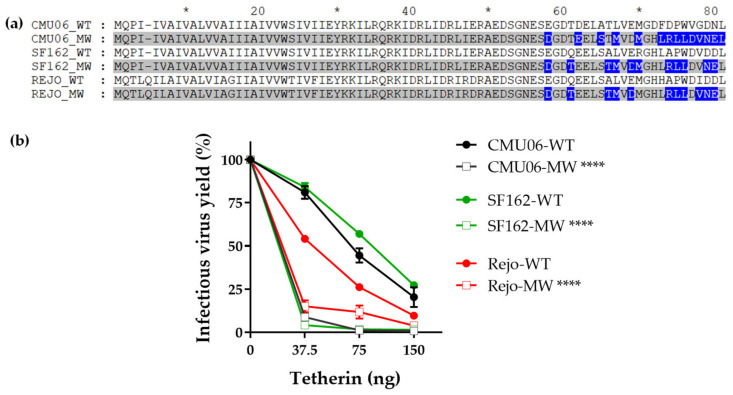
Effect of Vpu C-term swap on virus release in presence of tetherin. Vpu C-term MW965.26 was swapped in context of different isolates including CMU06 (acute), SF162 (chronic) and Rejo (T/F). (**a**) Alignment showing the differences in sequences. Sequences with swapped Vpu C-terminus are shaded in gray and the difference between the MW and WT sequences are highlighted in blue, gaps are indicated by trace. *, denotes blocks of 10. (**b**) HEK293T cells were co-transfected with IMCs and various amounts of plasmid expressing human tetherin. Forty-eight hours post-transfection supernatants were tested for infectious virus release by infecting TZM.bl cells as in Figure 2. ****, *p* < 0.0001 by ANOVA compared to their respective WT.

Since swapping MW significantly impacted virus release in presence of tetherin (Figure 3a) we next examined the effect of MW VEOR in the context of other HIV-1 isolates. Results show that regardless of the number of amino acid changes between the WT and C-term swapped Vpu (Figure 5a), chimeric MW Vpu in all instances reduced the virus yield compared to their respective WT (Figure 5b).

In addition to Vpu (as C-terminus) the swapped VEOR also affects Env (as Env-SP). Our published data have shown that SP residues can affect the Env glycosylation and Env functions [4,30]. Thus, we next present the effect of VEOR on Env (as SP).

### 3.2. VEOR Impacts Exposure of Env Epitopes

**Figure 6:**
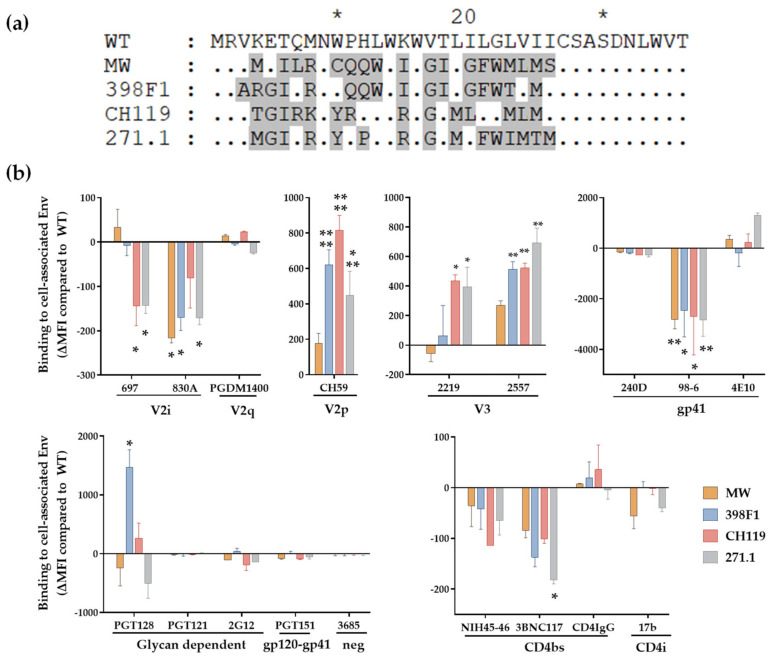
Effect of SP swap on CMU06 Env-antibody interaction. (**a**) Alignment showing the differences in SP sequences used to express CMU06 Env. SP sequences different from CMU06 native WT SP are shaded in gray and the similarities between the SP are indicated by dot, * denote blocks of 10. (**b**) Binding of mAbs to WT vs. SP-swapped Envs expressed on the surface of HEK293T cells. The mAbs were tested at following concentrations: 697, 830, and 3685 at 100 μg/mL; 2G12 at 50 μg/mL; PGDM1400, 2219, 2557, 240D, 98-6 and PGT121 at 25 μg/mL; 4E10, 17b at 10 μg/mL; PGT128, PGT151, 3BNC117 and CD4-IgG at 5 μg/mL; CH59 and NIH45-46 at 2.5 μg/mL. Normalized geometric mean fluorescence intensity (MFI) compared to WT (set as 100%) and SD from duplicates in one experiment are shown. MFI of negative control mAb were subtracted. Data were analyzed by ANOVA (*, *p* < 0.05; **, *p* < 0.01; *** *p* < 0.001; ****, *p* < 0.0001 vs. WT).

Given that VEOR acts as signal peptide (SP) in context of the Env glycoprotein we examined its implications on Env. We have shown that SP swap did not alter the Env incorporation into the virions (WT vs. swapped Env) (Figure 2b) [30]. However, SP altered the exposure of Env epitopes as revealed by a panel of monoclonal antibodies (mAbs) that target different Env regions. We transfected HEK293T cells with chimeric full length gp160 clones and 24 h post-transfection measured the binding of Env expressed on the cell surface to mAbs. Native CMU06 Env expressed with heterologous SP displayed differential binding pattern to most mAbs that were tested compared to CMU06-WT (Figure 6) and [30]. All 4 chimeric Envs showed altered binding to anti-Env mAbs including those targeting the V1V2, V3, gp41 and CD4bs. Binding of V2q mAb PGDM1400 was comparable suggesting similar Env levels and no deleterious effect on Env trimers. Binding of V3 crown mAbs (2219 and 2557) was enhanced while gp41-specific mAb (98–6) had reduced reactivity with all four SP-swaps vs. WT. Considering that the viruses had identical gp120 and gp41 coding sequence, the data strongly indicate that SP residues are associated with altering Env antigenicity.

**Figure 7:**
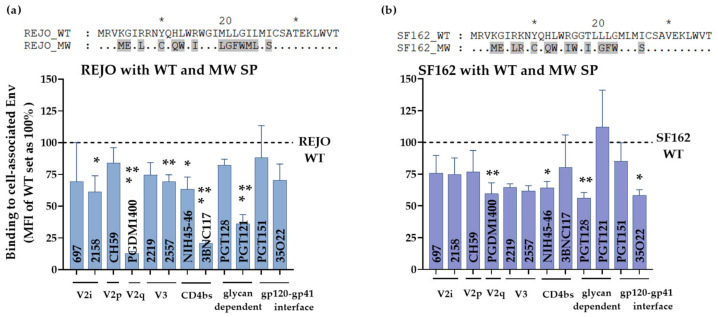
Effect of SP swap on REJO and SF162 Env-antibody interaction. HEK293T cells transfected with REJO (**a**) and SF162 (**b**) gp160 were analyzed for binding to mAbs targeting different Env epitopes. The mAbs were tested at following concentrations: 697, 830, and 3685 at 100 μg/mL; 2G12 at 50 μg/mL; PGDM1400, 2219, 2557, 240D, 98-6 and PGT121 at 25 μg/mL; 4E10, 17b at 10 μg/mL; PGT128, PGT151, 3BNC117 and CD4-IgG at 5 μg/mL; CH59 and NIH45-46 at 2.5 μg/mL. Normalized geometric mean fluorescence intensity (MFI) compared to WT (set as 100%) and SD from duplicates in one experiment are shown. MFI of negative control mAb were subtracted. Data were analyzed by ANOVA (*, *p* < 0.05; **, *p* < 0.01; *** *p* < 0.001; vs. WT). Sequence differences among the respective WT and swapped SP are also shown. Similar residues are shown as dot, * denote blocks of 10.

To evaluate if MW SP inflicts similarly changed exposure of epitopes in context of other Envs we tested the same panel of mAbs against REJO and SF162 Env expressed on HEK293T cell surface (Figure 7). Both REJO and SF162 Env with MW SP were recognized by mAbs to variable efficiency. Notably, MW SP with REJO Env almost abrogated the binding of V2q mAb PGDM1400 (Figure 7a) while binding of SF162 was reduced by 40% vs. their respective WTs (Figure 7b). The mAb PGDM1400 recognizes trimeric, cleaved Env [59]. Binding of CD4bs mAb 3BNC117 to REJO-MW was also reduced by 75% vs. REJO WT. These data suggest that chronic SP (MW) in the context of T/F isolate (REJO) negatively impacts the trimeric Env conformation. Of note, MW SP has glutamine (Q 12) at position 12. Position 12 in the envelope SP is enriched for histidine in transmitting and acute isolates suggesting a novel role for the SP in regulating envelope properties [60,61]. Notably, MW SP decreased the expression of Env only when in context of REJO (clade B, T/F) but not with other isolates such as JRFL (clade B, chronic) [4], CMU06, and SF162 [30]. Importantly, the Env protein backbone of REJO-WT and REJO-MW is identical.

HIV-1 Env is heavily glycosylated and glycans play an important role in modulating the epitope exposure. Interestingly, SP residues can alter Env glycosylation by modulating the relative amount of oligomannose and complex glycans which in turn can alter the Env-mAb interaction [4,30]. The PGT121 and PGT128 mAbs target the high-mannose patch centered around the glycan at position 332 on HIV Env [23]. Swapping the VEOR reduced the binding of all three Env expressed with MW SP while 398F1 significantly increased the binding of CMU06 to PGT128. Thus, VEOR as Env SP plays an important role in immune evasion by regulating the masking/exposure of its epitopes to host mounted antibodies.

### 3.3. SP Swaps Alter Env Glycosylation Affecting Virus Transmission via DC-SIGN

**Figure 8:**
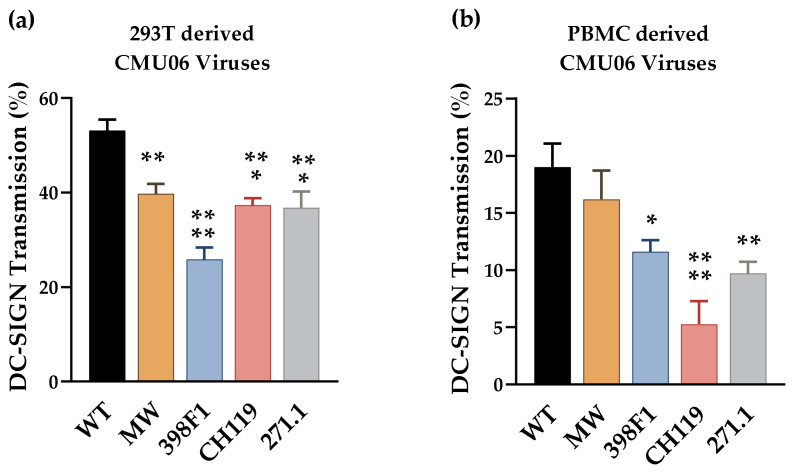
DC-SIGN mediated HIV-1 transmission. Raji–DC-SIGN+ cells were incubated for 2 h with WT or SP swapped viruses produced in (**a**) HEK293T and (**b**) human peripheral blood mononuclear cells (PBMC). Cells were washed to remove unbound viruses and added to CD4+ TZM.bl cells. Viral transmission to the TZM-bl cells was determined by luciferase activity and calculated based on infection in TZM.bl cells without Raji cells as control (set to 100%). Background luciferase activity was determined in co-cultures without any virus. *, *p* < 0.05; **, *p* < 0.01; ***, *p* < 0.001; ****, *p* < 0.0001 vs. by ANOVA.

The VEOR affected the glycosylation of all three Env as evident by the altered recognition of Env by mAbs whose binding depends on the N-linked glycans (Figure 6 and Figure 7) [30]. The composition of N-linked glycans on Env may also affect its interaction with cellular lectins and contribute to HIV-1 pathogenesis and transmission. In such case viruses produced with swapped SPs will be transmitted differentially via DC-SIGN (dendritic cell-specific ICAM-3-grabbing non-integrin; CD209). DC-SIGN is a C-type lectin which recognizes specific types of fucosylated glycans and subsets of oligomannose-type N-glycans [62]. Expressed on dendritic cells (DCs), DC-SIGN plays a key-role in the dissemination of HIV-1 by DCs. DC-SIGN captures HIV-1 at sites of entry, enabling virus transport to lymphoid tissues, where DC-SIGN efficiently transmits low amounts of HIV-1 to T cells. Since transmission via DC-SIGN relies on Env glycosylation which can also vary based on the cells used to produce the virus [63,64] we tested viruses that were produced in HEK293T and in human peripheral blood mononuclear cells (PBMC) (Figure 8).

Transmission of all four VEOR swapped CMU06 viruses produced in 293T cells was reduced significantly vs. the WT (Figure 8a). Similarly, reduced transfer of all 4 chimeric viruses produced in PBMC’s was observed (Figure 8b). The reduced transmission of swapped viruses was not associated with Env incorporation, as the levels of Env incorporated into these viruses, measured by Env/p24 ratios, was similar to WT (Figure 2b) [30]. The relatively different pattern observed between the viruses produced from different cell types, highlight the influence of changed glycan content which in turn is host-cell dependent [65,66,67,68].

## 4. Discussion

Progression of HIV-1 infection depends on the complex interplay between the virus-host interactions [69,70]. The selective pressure exerted by HIV-1 immune responses during infection results in ever-evolving virus variants with mutations (adaptations) that enable the virus to escape this response. HIV-1 accessory protein Vpu plays an important role in release of progeny virions and virus dissemination. Vpu and Env are expressed from the same bicistronic mRNA [70] with the 3′-end of Vpu gene overlapping with the N-terminus of Env gene (i.e., signal peptide). This study was undertaken to understand the in vitro physiological relevance of Vpu Env overlapping region (VEOR) in the context of the two viral proteins (Vpu and Env). We show that VEOR can significantly affect the ability of Vpu to counteract tetherin antagonism important for the release and spread of HIV-1, while simultaneously altering Env properties such that it can avoid recognition by the host mounted antibodies. This change in virus phenotype, as a function of VEOR, cannot be fully explained by the changes in the protein expression levels (Vpu) alone and highlights an additional strategy that may be employed by HIV-1 to evade host immune response.

While it is well established that Env incorporates changes due to the immune pressure exerted by the host, this overlapping region is also under selective immune pressure. Study by da Silva et al. [13] reported deletions of neutral and basic residues in the Env SP in viruses from early stages and insertion of basic residues in the hydrophobic region in late-stage isolates. Similarly, mutations in the Env SP were also reported when Env sequences isolated from transmission pairs were analyzed [71,72]. The overall hydrophobicity and number of basic residues in a SP are two critical determinants for HIV Env processing and glycosylation [73,74,75]. A higher degree of variability is observed among the Env SP of chronic Env as compared to T/F or acute Env of the same subtype. Further, comparison of transmitted sequences sampled very early in the infection and those during chronic infection revealed a histidine (His) enrichment at position 12 in early isolates and a recurring loss of this His residue in chronic isolates [60,61,76]. Thus, Env SP accrues changes as the HIV disease progresses and these changes accumulated in the SP during virus evolution implicate a potential role of the SP in immune evasion. Perhaps by means of Env-SP HIV-1 controls the expression of its Env and glycosylation that favors its sustained infection.

The swaps tested in this study altered the CMU06 Env phenotypically. Moreover, the glycosylation changes in the Env inflicted by changes in SP residues not only affected Env-antibody recognition [4,30], but also interaction with the host lectin DC-SIGN believed to facilitate virus spread from the mucosal entry site. It is not known if SP of T/F isolates offer any advantage to virus spread and dissemination, glycosylation of T/F viruses differ substantially from chronic strains [77,78,79]. The Env SP directs the nascent polypeptide to the ER where it folds, glycosylates and trimerizes in association with molecular chaperones (e.g., calnexin (CNX), calreticulin (CALR) etc.) [80,81,82]. Once the nascent polypeptide attains its native folding state the SP is cleaved and the native protein egresses the ER and translocate to the Golgi apparatus [15,81,82,83]. Further maturation of the glycans from oligomannose to hybrid and complex type occurs in the Golgi and depends on the compactness of Env folding, which consequently imposes or releases structural constrains to enzymes that generate hybrid or complex glycans in the Golgi. SP plays an important role in Env folding [14,15,80,81,84,85,86] and subtle changes in the Env folding will influence the remodeling of the N-linked glycans in the Golgi, creating a high structural diversity of Env N-linked glycans. Perhaps maintaining polymorphism in this Vpu-SP region (VEOR) is one of the mechanisms exploited by HIV-1 to alter its glycan cloak. Nevertheless, these results implicate Env SP in modulating the Env properties that assist the virus in evading the host-mounted immune response.

Here, we also show that changes in SP also impact important functions of Vpu protein. Vpu antagonizes tetherin by altering the trafficking of newly synthesized/recycling tetherin, through a complex interplay with host components, thereby decreasing the resupply of tetherin to the cell surface [87,88,89]. This disruption of tetherin trafficking is dependent on a sorting motif (59-ExxxLV-64) in a clathrin-dependent fashion [51] which in turn is regulated by the conserved aa 51-DSGxxS-56 motif. The DSGxxS motif among all Vpu tested here is identical however, swapping VEOR incurs changes in the motif ExxxLV. Exchange of MW VEOR abrogates Vpu’s ability to counteract tetherin vs. WT. Notably, amino acid changes similar to MW are introduced in the ExxxLV motif when VEOR of 271.1 was swapped, thus, these changes cannot explain the differences in the tetherin antagonism and virion release pattern observed among MW vs. 271.1 vs. WT. While 271.1 expresses both the upper and lower band similar to WT, only lower Vpu band is detected in MW Vpu suggesting that this lower molecular weight Vpu protein is inefficient in antagonizing tetherin. Further studies to understand the relevance of different sequences on host-virus interaction remains to be determined.

## 5. Conclusions

This study shows that polymorphisms in the VEOR have direct implications on HIV-1 infection. This overlapping region (1) regulate Vpu functions and Vpu-host interactions, facilitating virus replication and infection establishment, (2) impact Env glycosylation altering Env interaction with antibodies as Env-SP, facilitating immune evasion [4,30], and virus transmission via DC-SIGN. Thus, by incorporating changes in this region the virus uses it as another mechanism for immune evasion as any gain/loss in Vpu function to counteract tetherin/CD4 can also affect the surface-concentration of the viral Env on the cell, altering the susceptibility to immune surveillance by host-mounted Abs [90,91,92]. Studies to further understand the involvement of this region and its implication on HIV-1 pathogenesis and immune evasion are warranted.

## Data Availability

All available data are presented in the article and is available in [30].
